# NOWHERE ELSE TO GO: EFFECTIVENESS OF THE PASRR PROGRAM TO MEET THE NEEDS OF RESIDENTS WITH SMI ADMITTED TO NURSING HOMES

**DOI:** 10.1093/geroni/igac059.2366

**Published:** 2022-12-20

**Authors:** Taylor Bucy, Tetyana Shippee, Mark Woodhouse, John Bowblis, Shekinah Fashaw-Walters, Nathan Shippee

**Affiliations:** University of Minnesota School of Public Health, Minneapolis, Minnesota, United States; University of Minnesota, Minneapolis, Minnesota, United States; University of Minnesota, Minneapolis, Minnesota, United States; Miami University, Oxford, Ohio, United States; University of Minnesota School of Public Health, Minneapolis, Minnesota, United States; University of Minnesota School of Public Health, Minneapolis, Minnesota, United States

## Abstract

The number of adults with serious mental illness (SMI) who receive care in nursing homes (NHs) continues to rise. The Preadmission Screening and Resident Review (PASRR) program requires screening for SMI prior to NH placement, in order to avoid inappropriate admission and unnecessary institutional care. We interviewed staff responsible for the processing of PASRR documentation at four NHs in Minnesota (n=15), and obtained and analyzed all completed PASRR-II assessments in Minnesota from 2019 (N=532). PASRR assessments overwhelmingly recommended 24-hour NH care (94.7%) with 94% of assessments indicating a need for mental health services while at the NH. Most NH staff interviewed noted that PASRR is not used in the care planning process and described PASRR as a regulatory hoop. Staff shared that PASRR assessments could provide insight into an individual’s mental health history, current and future needs, and can be helpful in assessing NH capacity to provide such services. Although mental health services provided while at the NH are supposed to be facilitated in partnership with the county, there is a lack of follow-up and NH staff are largely left to deal with SMI in isolation. PASRR assessments are supposed to be a tool for care coordination, but leave the NH as the sole responsible point of contact for residents with SMI. A more integrated PASRR program that better focuses on incorporating PASRR into care planning and mental health service delivery in NHs and the broader community is necessary to improve the lives of individuals with SMI.

